# Rights and Lefts

**Published:** 1891-06

**Authors:** 


### Rights and Lefts.

Dr. Louis Jobert has published a work on the cause and frequency of left handedness. No purely left-handed race has ever been discovered, although there seems to be a difference in different tribes. Seventy per cent. of the inhabitants of the Pendjab use the left hand by preference, and the greater number of the Hottentots and Bushmen of South Africa use the left hand in preference to the right. Dr. Marro, as a result of his study of criminals, has found that from 14 to 22 per cent. of those who have been convicted of crime were left-handed, the highest ratio among people of all classes being only nine in the hundred.—[*Exchange.*

